# Comparing extended versus standard time window for thrombectomy: caseload, patient characteristics, treatment rates and outcomes—a prospective single-centre study

**DOI:** 10.1007/s00234-020-02531-8

**Published:** 2020-09-15

**Authors:** Bence Gunda, Ildikó Sipos, Rita Stang, Péter Böjti, Levente Dobronyi, Tímea Takács, Tamás Berényi, Balázs Futácsi, Péter Barsi, Gábor Rudas, Balázs Kis, István Szikora, Dániel Bereczki

**Affiliations:** 1 Department of Neurology, Semmelweis University, Budapest, Hungary; 2 Department of Emergency Medicine, Semmelweis University, Budapest, Hungary; 3 Department of Radiology, Medical Imaging Centre, Semmelweis University, Budapest, Hungary; 4 Department of Neuroradiology, Medical Imaging Centre, Semmelweis University, Budapest, Hungary; 5 National Institute of Clinical Neurosciences, Budapest, Hungary; 6 MTA-SE Neuroepidemiological Research Group, Budapest, Hungary

**Keywords:** Acute ischemic stroke, Thrombectomy, Extended time window, Caseload, Patient selection

## Abstract

**Purpose:**

New guidelines recommend thrombectomy up to 24 h in selected patients; however, the workload and benefit of extending time window are not known. We conducted a prospective single-centre study to determine the caseload, imaging and interventional need of extended time window.

**Methods:**

All consecutive ischemic stroke patients within 24 h from onset in an 11-month period were included. Thrombectomy eligibility in the 0–6 h time window was based on current guidelines; in the 6–24 h time window, it was based on a combination of DEFUSE 3 and DAWN study criteria using MRI to identify target mismatch. Clinical outcome in treated patients was assessed at 3 months.

**Results:**

Within 24 h of onset, 437 patients were admitted. In the 0–6 h time window, 238 patients (54.5%) arrived of whom 221 (92.9%) underwent CTA or MRA, 82 (34.5%) had large vessel occlusion (LVO), 30 (12.6%) had thrombectomy and 11 (36.6%) became independent (mRS ≤ 2). In the extended 6–24 h time window, 199 patients (45.5%) arrived of whom 127 (63.8%) underwent CTA or MRA, 44 (22.1%) had LVO, 8 (4%) had thrombectomy and 4 (50%) became independent.

**Conclusion:**

Extending the time window from 6 to 24 h results in a 26.7% increase in patients receiving thrombectomy and a 36.4% increase of independent clinical outcome in treated patients at the price of a significantly increased burden of clinical and imaging screening due to the similar caseload but a smaller proportion of treatment eligible patients in the extended as compared with the standard time window.

**Electronic supplementary material:**

The online version of this article (10.1007/s00234-020-02531-8) contains supplementary material, which is available to authorized users.

## Introduction

New AHA [1] and ESO [2] guidelines recommend endovascular treatment (EVT) of large vessel occlusion (LVO) strokes in an extended time window of 6 to 24 h in patients selected with advanced imaging, based on DAWN [3] and DEFUSE 3 [4] trials. However, there are no published data from these trials and only retrospective and speculative data from national registries or single centres [5–9] on the burden of screening these patients, their treatment rates and outcomes as compared with those in the standard time window within 6 h.

## Aims

We conducted a prospective single-centre study to determine the caseload, imaging and interventional need of acute stroke management in the extended as compared with the standard time window.

## Methods

All consecutive ischemic stroke patients admitted within 24 h from onset to a large university hospital emergency room (ER) from 01 February 2019 to 31 December 2019 were included. Implementing the new guideline recommendations, Emergency Medical Services protocol required all acute stroke patients within 24 h to be urgently transported to our stroke centre and ER also triaged these patients as critical. In the 0–6 hour time window, all patients regardless of clinical severity were considered for treatment. Patients with occlusion of the internal carotid artery (ICA) and/or middle cerebral artery (MCA) M1 segment with ASPECTS ≥ 6, or MCA M2 segment, ACA A1 segment, PCA P1 segment or basilar artery were eligible for EVT. In the 6–24 h time window, only patients with NIHSS ≥ 6 or fluctuating/brainstem symptoms and premorbid mRS ≤ 2 underwent CTA, and only those with ICA, M1 and basilar occlusions were regarded as potentially treatable LVO and underwent MRI to identify target mismatch. Patients with unknown onset strokes recognised within 4 h had primary MRI. EVT eligibility was determined by DEFUSE-3 criteria between 6-16 h and simplified DAWN criteria between 16 and 24 h. See our detailed protocol in Supplementary Figure. Time window, age, stroke severity (NIHSS), non-invasive angiography use, presence of LVO and EVT use were assessed. Clinical outcome (mRS) in the subset of treated patients was assessed at 3 months. Patients in the 0–6 and 6–24 time windows were compared with appropriate statistical tests.

## Results

**Fig. 1:**
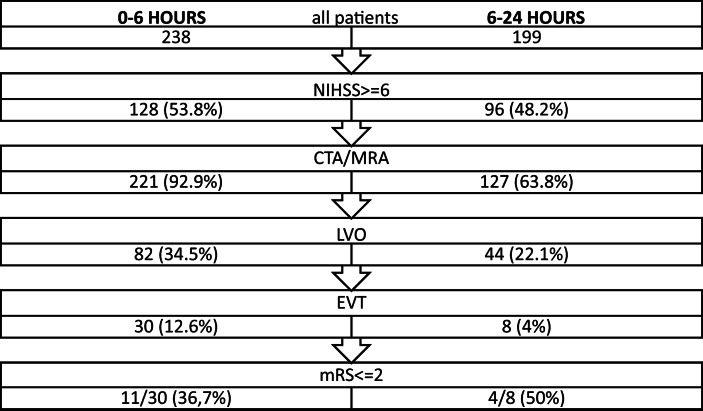
Flowchart in standard and extended time windows

In this 11-month period, 437 ischemic stroke patients were admitted within 24 h of onset (Fig. 1). Two hundred thirty-eight patients (54.5%) arrived in the standard 0–6 hour time window of whom 221 (92.9%) underwent CTA or MRA, 82 (34.5%) had LVO, 30 (12.6%) had EVT of whom 11 (36.6%) became functionally independent (mRS < =2). Number needed to screen (NNS) to find one EVT eligible patient was 8.

One hundred ninety-nine patients (45.5%) arrived in the extended 6-24 h time window of whom 127 (63.8%) underwent CTA or MRA, 44 (22.1%) had LVO, 8 (4%) had EVT of whom 4 (50%) became functionally independent (mRS < =2). NNS to find one EVT eligible patient was 25.

Patients in the two time windows were comparable in numbers, with similar age and sex ratio (Table 1). Patients in the standard time window had more severe strokes (median NIHSS 6 vs 5; *p* = 0.011), had LVO more often (34.5 vs 22.1%; *p* = 0.0046), were eligible for EVT more often (12.6 vs 4%; *p* = 0.001), and had greater proportion of EVT eligibility in case of LVO (36.6 vs 18.2%, *p* = 0.0415). Treated patients in the two time windows had similar age, but those in the standard time window had more severe strokes (median NIHSS 15 vs 8) and worse clinical outcome (median mRS 4 vs 2.5) not reaching statistical significance because of low numbers (Table 2). 65.1% of LVO strokes and 78.9% of EVT eligible patients were in the 0–6 hour time window. The extension of time window translated into an 83.6% increase in patient numbers for emergency clinical screening, a 57.5% increase in non-invasive angiography, a 26.7% increase in EVT and a 36.4% increase of independent clinical outcome in treated patients.

**Table 1:** Comparison of all patients in standard and extended time windows

	0–6 h	6–24 h	*p*
Number of cases	238	199	n.a.
Male (%)	50	49.7	0.96 (chi-square)
Age (mean, SD)	70.5 (± 12.4)	70.9 (± 11.7)	0.744 (*t* test)
NIHSS (median, IQR)	6 (4–12)	5 (3–8)	0.011 (Mann-Whitney)
CTA/MRA (%)	92.9	63.8	< 0.001 (chi-square)
LVO (%)	34.5	22.1	0.0046 (chi-square)
EVT (%)	12.6	4	0.001 (Fisher)
EVT in LVO (%)	36.6	18.2	0.0415 (Fisher)
NNS for EVT	8	25	n.a.

**Table 2:** Comparison of treated patients in standard and extended time windows

	0–6 h	6–24 h	*p*
Number of EVTs	30	8	n.a.
Age (mean, SD)	68.2 (± 12.2)	69.1(± 14.4)	0.855 (t test)
NIHSS (median, IQR)	15 (9–18)	8 (5–15)	0.086 (Mann-Whitney)
mRS (median, IQR)	4 (1–6)	2.5 (0–5)	0.44 (Mann-Whitney)

## Discussion

In our management environment implementing new guidelines on extended time window, 45.5% of all stroke patients within 24 h arrived beyond 6 h. This is a larger proportion than in retrospective studies from before current guidelines reporting 35% [6] and 33% [8].

In the 0–6 hour time window, the rate of LVO was high (34.5%) and the rate of EVT (12.6%) was higher than the post hoc calculated EVT eligibility (10.5%) from a single-centre registry with similar selection criteria, but before the evidence-based thrombectomy era (2003–2014) [8].

In the 6–24 hour time window, the rate of LVO (22.1%) was similar, but the rate of EVT (4%) was lower in our study than in a retrospective analysis [7] (19.6% and 9.2% respectively) using similar DAWN + DEFUSE 3 criteria. However, data on patients managed outside trial criteria and not referred to the tertiary centre were not available for comparison; therefore, we expect selection bias towards LVO patients more likely to be eligible for intervention. EVT rate in our study was comparable to eligibility estimates by Lee et al (3.6%) [6].

EVT rate in our patients was significantly lower in the extended than in the standard time window (4 vs 12.6%). This is due to both a lower rate of LVO (22.1 vs 34.5 %) and a lower rate of EVT in LVO strokes (18.2 vs 36.6%). Lower rate of LVO may be explained by the following: (1) a lower rate of non-invasive angiography (63.8 vs 92.2%) as mild strokes (NIHSS < 6) were not candidates for CTA; therefore, LVOs causing only mild symptoms may have been missed. (2) Stricter definition of treatment eligible LVO (only ICA, M1 and BA) and (3) the fact that more severe strokes with higher NIHSS and higher probability of LVO are more alarming and thus prompt earlier presentation at ER. Indeed, NIHSS was significantly lower in the late time group (median 5 vs 6, *p* = 0.011). The lower rate of EVT in LVO strokes is due to stricter imaging eligibility criteria beyond 6 h: higher ASPECTS, smaller core and demonstration of penumbra that rapidly decreases with time. EVT eligibility may improve if ongoing studies (TENSION, IN-EXTREMIS) show benefit with more relaxed selection criteria such as milder strokes and larger infarct cores; however, “time is brain” remains a valid and important concept on a population basis.

We found that treatment outcome was better in the extended than in the standard time window (median mRS 2.5 vs 4) that probably reflects both lower stroke severity (median NIHSS 8 vs 15) and stricter imaging criteria in this group; however, due to small numbers, these differences did not reach statistical significance. Independent clinical outcome (mRS ≤ 2) was seen in 50% of our treated patients in the 6–24 h group that is similar to the landmark studies (DAWN: 49%, DEFUSE 3: 45%) [3, 4].

Our study reports a larger burden of screening to find EVT eligible patients (NNS 25) than that of Jadhav et al. [7] (NNS 11) with similar selection criteria but in a pre-selected patient population. In our study, the extension of time window led to a slightly smaller increase in actually treated patients (26.7%) than the 33.3% increase of theoretically EVT eligible patients from a retrospective analysis of a single-centre registry [5]. Our results—in line with previous studies [5–7], and [9] comparing pre- and post-DAWN management—show that the main burden of extended time window lies on the clinical and imaging screening of patients (EMS, ER, neurologists and radiologists) rather than their treatment because of the smaller proportion of EVT eligible patients compared with the standard time window. This needs to be taken into account when planning stroke care pathways and resource allocation. However, a more than 25% increase in EVT rate is clinically important, and late thrombectomy up to 24 h was shown to be both clinically highly efficacious [3, 4] and cost-effective [10]. We also demonstrated good clinical outcomes in the late time window in 50% of patients similar to the landmark studies [3, 4]. Therefore, we recommend that patients in the extended time window should also be actively screened for thrombectomy.

We believe that our results more precisely reflect current real life situation than previous retrospective analyses because our data are based on a prospective study of unselected patients in a current guideline–driven stroke management system.

The main limitation of our study is its single-centre design and the small number of treated patients precluding firm conclusions on outcomes.

## Conclusion

Extending the time window from 6 to 24 h for thrombectomy results in a more than 25% increase in patients receiving thrombectomy at the price of a significantly increased burden of clinical and imaging screening due to the smaller proportion of treatment eligible patients beyond 6 h based on current strict eligibility criteria. However, late patients benefit at least equally from treatment; therefore, we recommend that they should also be actively screened. Further studies are needed to evaluate the benefit of thrombectomy beyond 6 h with less strict imaging and clinical criteria. Efforts should be made to achieve earlier patient arrival, because “time is brain” is still an important paradigm on a population level.

## Electronic supplementary material

ESM 1 (PDF 429 kb).
