# Supplementary material for: Comparing extended versus standard time window for thrombectomy: caseload, patient characteristics, treatment rates and outcomes—a prospective single-centre study
Source: Neuroradiology. 2020 Sep 15;63(4):603–7. doi: 10.1007/s00234-020-02531-8 (PMC7966226; doi:10.1007/s00234-020-02531-8)
Supplement: Supplementary file 1 — (PDF 429 kb). [file 234_2020_2531_MOESM1_ESM.pdf]

Comparing extended versus standard time window for thrombectomy: caseload, patient characteristics and treatment rates –a prospective single centre study

NEURORADIOLOGY

Gunda B[1], Sipos I[1], Stang R[1], Böjti P[1], Dobronyi L[1], Berényi T[2], Futácsi B[3], Barsi P[4], Rudas G[4], Kis B[5], Szikora I[5], Bereczki D[1,6]

1. Department of Neurology, Semmelweis University
2. Department of Emergency Medicine, Semmelweis University
3. Department of Radiology, Semmelweis University
4. MR Research Centre, Semmelweis University
5. National Institute of Clinical Neurosciences
6. MTA-SE Neuroepidemiological Research Group

Corresponding author: Bence Gunda, bence.gunda@gmail.com

Supplementary Figure. Acute stroke management protocol

Time (hours)

0-6 **CT+CTA**

IVT within 4,5 h if eligible

+EVT if: -anterior LVO (ICA, M1) and ASPECTS>=6, or

- M2, A1, P1, BA

6-24 **CT**

+CTA, if: -NIHSS>=6 or fluctuating or brainstem symptoms, and
-premorbid mRS<=2

+MR (PWI), if: -ICA, M1, BA occlusion, and
-no large hemispheric (>1/3 MCA) or brainstem hypodensity, ASPECTS >=7

EVT in anterior LVO, if mismatch present:

6-16 h (DEFUSE 3) core-penumbra: core<70ml, penumbra>15 ml and 80% of core

16-24 h (DAWN) core-clinical: NIHSS 10-19 and core<30ml or NIHSS>=20 and core<50ml

EVT in posterior LAO if no large brainstem DWI lesion, individual decision

Unknown onset (wake up)

symptom recognition **<4 h**: **MR**

IVT, if DWI-FLAIR mismatch

symptom recognition **>4 h**: **CT** with 0-6 or 6-24 h protocol based on time of LSW
